# Supplementary material for: Indication of frailty transitions on 2-year adverse health outcomes among older Chinese inpatients: Insight from a multicenter prospective cohort study
Source: PLoS One. 2025 Jan 8;20(1):e0313775. doi: 10.1371/journal.pone.0313775 (PMC11709267; doi:10.1371/journal.pone.0313775)
Supplement: S1 Table — (DOCX) [file pone.0313775.s001.docx]

Supplementary table 1 Prevalence of frailty across demographic groups (n [%])

| Characteristics | Overall | Frail | Pre-frail | Non-frail | *P* |
| --- | --- | --- | --- | --- | --- |
| Sample size | 8256 (100.00) | 1343 (16.27) | 3553 (43.04) | 3360 (40.70) |  |
| Gender |  |  |  |  | <0.01 |
| Female | 3506 (42.47) | 651 (18.57) | 1572 (44.84) | 1283 (36.59) |  |
| Male | 4750 (57.53） | 692 (14.57) | 1981 (41.71) | 2077 (43.73) |  |
| Age |  |  |  |  | <0.01 |
| 65-74 | 5880 (71.22) | 835 (14.20) | 2511 (42.70) | 2534 (43.10) |  |
| 75-84 | 2116 (25.63) | 440 (20.79) | 917 (43.34) | 759 (35.87) |  |
| ≥85 | 260 (3.15) | 68 (26.15) | 125 (48.08) | 67 (25.77) |  |
| Ethnicity |  |  |  |  | <0.01 |
| Han | 7772 (94.14) | 1203 (15.48) | 3338 (42.95) | 3231 (41.57) |  |
| Others | 484 (5.86) | 140 (28.93) | 215 (44.42) | 129 (26.65) |  |
| Education |  |  |  |  | <0.01 |
| Illiterate | 1313 (15.90) | 323 (24.60) | 571 (43.49) | 419 (31.91) |  |
| Primary school | 2389 (28.94) | 397(16.62) | 1014 (42.44) | 978 (40.94) |  |
| Middle school | 3349 (40.56) | 466 (13.91) | 1449 (43.27) | 1434 (42.82) |  |
| College or above | 1205 (14.60) | 157 (13.03) | 519 (43.07) | 529 (43.90) |  |
| Marital status |  |  |  |  | <0.01 |
| Married | 7329 (88.85) | 1143 (15.60) | 3150 (42.98) | 3036 (41.42) |  |
| Divorced or widowed | 920 (11.15) | 199 (21.63) | 400 (43.48) | 321 (34.89) |  |
| BMI |  |  |  |  | <0.01 |
| Underweight | 520 (6.37) | 149 (28.65) | 245 (47.12) | 126(24.23) |  |
| Normal weight | 3947 (48.36) | 648 (16.42) | 1745 (44.21) | 1554 (39.37) |  |
| Overweight | 2866 (35.11) | 386 (13.47) | 1188 (41.45) | 1292 (45.08) |  |

Supplementary table 1 Prevalence of frailty across demographic groups (n [%]) (Continued)

| Characteristics | Overall | Frail | Pre-frail | Non-frail | *P* |
| --- | --- | --- | --- | --- | --- |
| Obese | 829 (10.16) | 129 (15.56) | 341 (41.13) | 359 (43.31) |  |
| Surgery |  |  |  |  | <0.01 |
| No | 5467 (66.22) | 1058 (19.35) | 2474 (45.25) | 1935 (35.39) |  |
| Yes | 2789 (33.78) | 285 (10.22) | 1079 (38.69) | 1425 (51.09) |  |
| Smoking status |  |  |  |  | <0.01 |
| Non-smoker | 5447 (65.98) | 918 (16.85) | 2398 (44.02) | 2131 (39.12) |  |
| Current smoker | 943 (11.42） | 127 (13.47） | 385 (40.83） | 431 (45.71） |  |
| Former smoker | 1866 (22.60) | 298 (15.97) | 770 (41.26) | 798 (42.77) |  |
| Drinking |  |  |  |  | <0.01 |
| Non-drinker | 6326 (76.62) | 1080 (17.07) | 2750 (43.47) | 2496 (39.46) |  |
| Current drinker | 973 (11.79) | 99 (10.17) | 375 (38.54) | 499 (51.28) |  |
| Former drinker | 957 (11.59) | 164 (17.14) | 428 (44.72) | 365 (38.14) |  |
| Falls in the past year |  |  |  |  | <0.01 |
| NO | 7082 (85.78) | 1056 (14.91) | 3000 (42.36) | 3026 (42.73) |  |
| Yes | 1174 (14.22) | 287 (24.45) | 553 (47.10) | 334 (28.45) |  |
| Bedridden for ≥ 4 weeks |  |  |  |  | <0.01 |
| NO | 8059 (97.61) | 1215 (15.08) | 3500 (43.43) | 3344 (41.49) |  |
| Yes | 197 (2.39) | 128 (64.97) | 53 (26.90) | 16 (8.12) |  |
| polypharmacy |  |  |  |  | <0.01 |
| NO | 6324 (76.60) | 918 (14.52) | 2694 (42.60) | 2712 (42.88) |  |
| Yes | 1932 (23.40) | 425 (22.00) | 859 (44.46) | 648 (33.54) |  |
| Nutritional status |  |  |  |  | <0.01 |
| Normal nutrition | 4660 (56.44) | 346 (7.42) | 1787 (38.35) | 2527 (54.23) |  |

Supplementary table 1 Prevalence of frailty across demographic groups (n [%]) (Continued)

| Characteristics | Overall | Frail | Pre-frail | Non-frail | *P* |
| --- | --- | --- | --- | --- | --- |
| Malnutrition risk | 2793 (33.83) | 604 (21.63) | 1450 (51.92) | 739 (26.46) |  |
| Malnutrition | 803 (9.73) | 393 (48.94) | 316 (39.35) | 94 (11.71) |  |
| Depression |  |  |  |  | <0.01 |
| NO | 6808 (84.51) | 779 (11.44) | 2905 (42.67) | 3124 (45.89) |  |
| Yes | 1248 (15.49) | 508 (40.71) | 576 (46.15) | 164 (13.14) |  |
| Cognition |  |  |  |  | <0.01 |
| Normal cognition | 6264 (80.19) | 828 (13.22) | 2648 (42.27) | 2788 (44.51) |  |
| Cognitive impairment | 1547 (19.81) | 382 (24.69) | 702 (45.38) | 463 (29.93) |  |
| Handgrip strength |  |  |  |  | <0.01 |
| Normal | 4207 (50.96) | 990 (23.53) | 1893 (45.00) | 1324 (31.47) |  |
| Low-level | 4049 (49.04) | 353 (8.72) | 1660 (41.00) | 2036 (50.28) |  |
| Vision |  |  |  |  | <0.01 |
| Normal | 6439 (77.99) | 931 (14.46) | 2773 (43.07) | 2735 (42.48) |  |
| Abnormal | 1817 (22.01) | 412 (22.67) | 780 (42.93) | 625 (34.40) |  |
| Hearing |  |  |  |  | <0.01 |
| Normal | 6685 (80.97) | 977 (14.61) | 2900 (43.38) | 2808 (42.00) |  |
| Abnormal | 1571 (19.03) | 366 (23.30) | 653 (41.57) | 552 (35.14) |  |
| Sleep |  |  |  |  | <0.01 |
| Normal | 4720 (57.17) | 546 (11.57) | 1939 (41.08) | 2235 (47.35) |  |
| Abnormal | 3536 (42.83) | 797 (22.54) | 1614 (45.64) | 1125 (31.82) |  |
| Urinary |  |  |  |  | <0.01 |
| Normal | 7173 (86.88) | 1063 (14.82) | 3099 (43.20) | 3011 (41.98) |  |
| Abnormal | 1083 (13.12) | 280 (25.85) | 454 (41.92) | 349 (32.23) |  |

Supplementary table 1 Prevalence of frailty across demographic groups (n [%]) (Continued)

| Characteristics | Overall | Frail | Pre-frail | Non-frail | *P* |
| --- | --- | --- | --- | --- | --- |
| Defecation |  |  |  |  | <0.01 |
| Normal | 7314 (88.59) | 1061 (14.51) | 3102 (42.41) | 3151 (43.08) |  |
| Abnormal defecation | 942 (11.41) | 282 (29.94) | 451 (47.88) | 209 (22.19) |  |
| Tumor |  |  |  |  | <0.01 |
| No | 6179 (74.84) | 1079 (17.46) | 2614 (42.30) | 2486 (40.23) |  |
| Yes | 2077 (25.16) | 264 (12.71) | 939 (45.21) | 874 (42.08) |  |
| ADL |  |  |  |  | <0.01 |
| Barthel scale score > 60 | 7483 (90.64) | 1018 (13.60) | 3288 (43.94) | 3177 (42.46) |  |
| Barthel scale score 41-60 | 401 (4.86) | 165 (41.15) | 129 (32.17) | 107 (26.68) |  |
| Barthel scale score ≤ 40 | 372 (4.51) | 160 (43.01) | 136 (36.56) | 76 (20.43) |  |
| IADL | 8256 | 4.77±2.58 | 6.92±1.70 | 7.64±0.98 |  |
